# Septic Arthritis in Immunosuppressed Patients: Do Laboratory Values Help?

**DOI:** 10.5435/JAAOSGlobal-D-20-00007

**Published:** 2020-03-17

**Authors:** Jared Bell, Luke Rasmussen, Arun Kumar, Michael G. Heckman, Elizabeth R. Lesser, Joseph Whalen, Glenn G. Shi, Benjamin K. Wilke

**Affiliations:** From the Department of Orthopedic Surgery, Mayo Clinic, Jacksonville, FL.

## Abstract

Previous studies have recommended synovial fluid cell count thresholds of 50,000 cells/mm^−3^ to diagnose septic arthritis; however, data to support this are limited. It is also unknown if this value is valid in immunosuppressed patients.

**Methods:**

We retrospectively reviewed 33 immunosuppressed patients treated at our institution from 2008 to 2018. We compared culture-positive patients with culture-negative patients.

**Results:**

We found no statistically significant differences in synovial fluid cell count, percent synovial fluid neutrophils, erythrocyte sedimentation rate, or C-reactive protein between the groups (all *P* = 0.081). The median synovial fluid cell count in the culture-positive cohort was 29,000 cells/mm^−3^, with only 31.2% having >50,000 cells/mm^−3^.

**Conclusion:**

Traditional synovial fluid cell thresholds are not a reliable method of diagnosing septic arthritis in immunosuppressed patients.

There are approximately 20,000 cases of septic arthritis diagnosed annually in the United States.^1,2^ It is a diagnosis that can be associated with notable morbidity, including joint destruction, sepsis, and even increased mortality, made worse by a delay in treatment.^3,4,5,6,7,8^ Prompt diagnosis therefore becomes critical for reducing complications. Accurate diagnosis, however, may be challenging because several pathologies such as crystalline arthropathies or inflammatory arthropathies may present with a similar clinical picture.^9,10,11,12^

Septic arthritis has historically been diagnosed by combining clinical examination and laboratory data, with a positive culture remaining the benchmark for diagnosis. Waiting for the culture results however may require several days, which can lead to increased morbidity.^13^ Clinicians must therefore rely on testing that is obtained more rapidly but may not be specific for septic arthritis. Historically, these tests have included a complete blood count, erythrocyte sedimentation rate (ESR), C-reactive protein (CRP), synovial fluid cell count, and percent of synovial fluid neutrophils (% neutrophils). Previous studies have suggested the use of a synovial fluid cell count of >50,000 cells/mm^−3^ from a native joint aspiration as the threshold for the diagnosis of septic arthritis.^9,10,14,15,16,17^ The addition of serum white blood cells (WBC) >11.0 × 10^9^/L and ESR >30 mm/hr with this synovial fluid cell count threshold has been reported to have a sensitivity approaching 100%.^18^

Despite the near-dogmatic approach to the 50,000 cell cutoff, the evidence to support this value is limited. In addition, it is unknown if patients in an immunosuppressed state can mount an effective immune response to trigger this cutoff or if a lower value should be used for these patients to avoid a delayed or missed diagnosis.^10,19–21^ The purpose of this study was to review our immunosuppressed patients who had a diagnosis of septic arthritis to determine whether 50,000 cells/mm^−3^ is a reliable cutoff for this patient population.

## Methods

Institutional Review Board approval was obtained for this retrospective review. The hospital medical database was then queried using ICD codes to identify 490 patients with a diagnosis of septic arthritis between February 2008 and August 2018. Patient charts were then manually reviewed to exclude patients who did not undergo an aspiration, those with a history of a joint replacement involving the joint in question, and those who had a negative aspiration but had been given antibiotics before the aspiration. We additionally excluded any patient without a diagnosis of immunodeficiency. For the purposes of our study, we defined immunodeficiency as any solid organ transplant patient on antirejection medication, a patient with cancer who was receiving neutropenic chemotherapy at the time of diagnosis of septic arthritis, a patient with an inflammatory arthropathy who was receiving immunotherapy at the time of diagnosis, or a patient with end-stage renal or liver failure. Patients were included if they had a diagnosis of immunodeficiency and had culture-positive septic arthritis in a native joint. We additionally included patients who had undergone an aspiration with negative culture results before administration of antibiotics to serve as a comparison group. Thirty-three patients were identified based on these criteria and were included in the study: 16 with positive cultures and 17 with negative cultures.

Data were collected including patient age, sex, immunosuppression diagnosis, antibiotic administration before aspiration, affected joint, laterality, and if the patient underwent surgical débridement of the joint. Serum laboratory values that were recorded included WBC count, ESR, and CRP. Synovial laboratory values recorded included synovial fluid cell count and percentage of synovial neutrophils. Culture results and species differentiation were also documented.

The cohort included 19 men (57.5%) and 14 women (42.5%). The median age was 66 years (range: 21 to 89 years). Seventy-six percent of the cohort's affected joint involved the knee, but other affected joints included the shoulder, wrist, and hip. Patients with cancer on neutropenic chemotherapy and solid organ transplant patients on antirejection medication accounted for most of the cohort's immunosuppression diagnosis, but other diagnoses included rheumatoid disease, renal failure, and liver failure. Patient characteristics are summarized in Table 1.

**Table 1:** Patient Characteristics

	Negative Culture (N = 17)	Positive Culture (N = 16)	*P* Value
Age at diagnosis	71 (21, 89)	59 (36, 84)	0.12
Males	7 (41.2%)	12 (75.0%)	**0.049**
Affected joint (knee/hip/shoulder)			0.17
Hip	0 (0.0%)	1 (6.2%)	
Knee	13 (76.5%)	12 (75.0%)	
Shoulder	1 (5.9%)	3 (18.8%)	
Wrist	3 (17.6%)	0 (0.0%)	
Immunosuppression diagnosis			
Chemotherapy	5 (29.4%)	6 (37.5%)	
Rheumatoid disease	5 (29.4%	1 (6.2%)	
Organ transplantation	7 (41.2%)	4 (25.0%)	
Renal failure	0 (0.0%)	3 (18.8%)	
Liver failure	0 (0.0%)	2 (12.5%)	
Right laterality	11 (64.7%)	9 (56.2%)	0.62
Treated surgically	6 (35.3%)	16 (100.0%)	**<0.001**
Received antibiotics before aspiration	0 (0.0%)	4 (25.0%)	**0.028**
Culture specimen			N/A
*Staphylococcus aureus*	N/A	5 (31.2%)	
*Streptococcus* species	N/A	5 (31.2%)	
*Pseudomonas aeruginosa*	N/A	3 (18.8%)	
Other species	N/A	3 (18.8%)	

Continuous variables are summarized with median (range), and categorical variables are summarized with number (%). *P* values less than 0.05 are considered statistically significant and are shown in bold.

Continuous variables were summarized with median and range, and categorical variables with frequency and percent. Continuous variables were compared between patients with a positive culture and those with a negative culture using a Wilcoxon rank-sum test, whereas categorical variables were compared between these two groups using a Pearson chi-square test. To evaluate the ability of % neutrophils, ESR at diagnosis, CRP at diagnosis, WBC at diagnosis, and synovial fluid cell count from aspiration to discriminate between patients with a positive culture and those with a negative culture, we estimated the area under the ROC curve (AUC) along with 95% confidence intervals (CIs). An AUC of 1.0 indicates perfect predictive ability, whereas and AUC of 0.5 indicates predictive ability equal to chance. All statistical tests were two-sided, and *P*-values less than 0.05 were considered statistically significant. If patients had multiple aspirations performed, only the first was included to satisfy the statistical assumption of independence. All statistical analyses were performed in R statistical software (version 3.4.2; R Foundation for Statistical Computing).

## Results

There were 33 patients included in the final analysis: 16 (48.5%) had a positive synovial fluid culture and 17 (51.5%) had a negative culture. There was a higher proportion of men in the positive culture cohort compared with the negative culture cohort (75% versus 41.2%, *P* = 0.049), and although not statistically significant, the positive culture cohort tended to be younger (median: 59 versus 71 years, *P* = 0.12). As expected, antibiotic use before aspiration and surgical treatment differed between the two groups (Table 1). No other patient characteristic differences were observed. All 16 patients in the culture-positive cohort (100.0%) underwent surgical débridement. Six patients in the culture-negative cohort (35.3%) underwent surgical débridement based on high clinical suspicion for septic arthritis. Most of the culture-positive cohort speciation included *Staphylococcus aureus* and *Streptococcus* species (62.5%). Other species identified in this study included *Pseudomonas aeruginosa* (18.8%), *Candida zeylanoides* (6.2%), *Laceyella sacchari* (6.2%), and *Mycobacterium chelonae* (6.2%) (Table 1).

**Figure 1:**
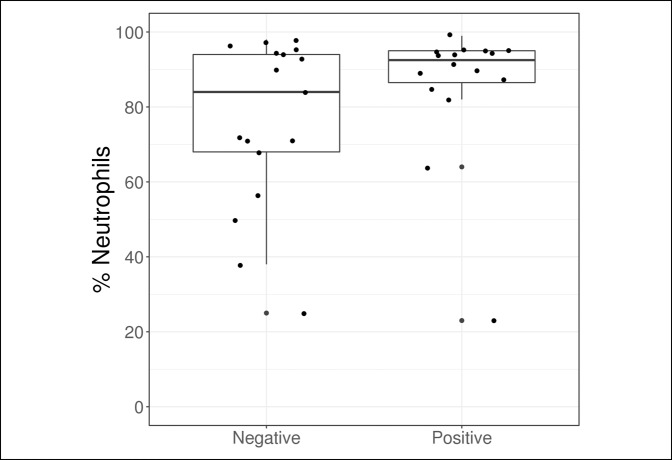
Boxplot of synovial fluid % neutrophils according to the culture result.

**Figure 2:**
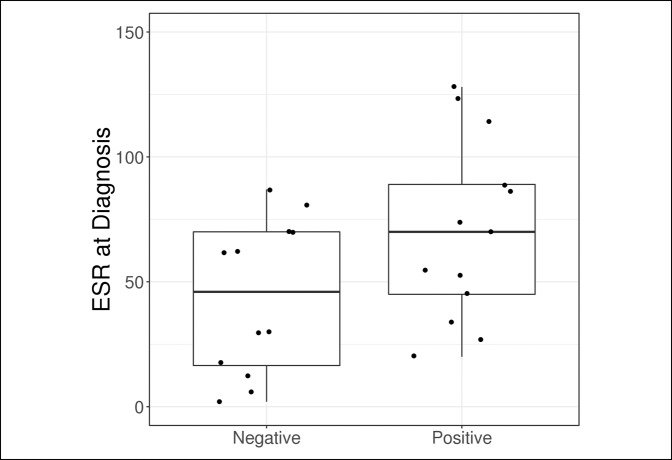
Boxplot of ESR according to the culture result. ESR = erythrocyte sedimentation rate

**Figure 3:**
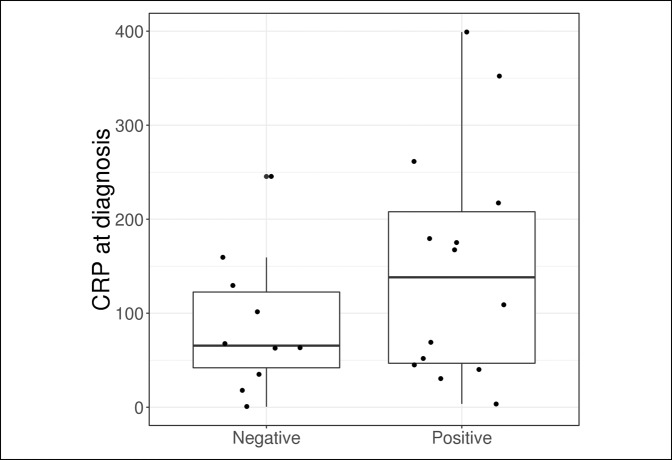
Boxplot of CRP according to the culture result. CRP = C-reactive protein

**Figure 4:**
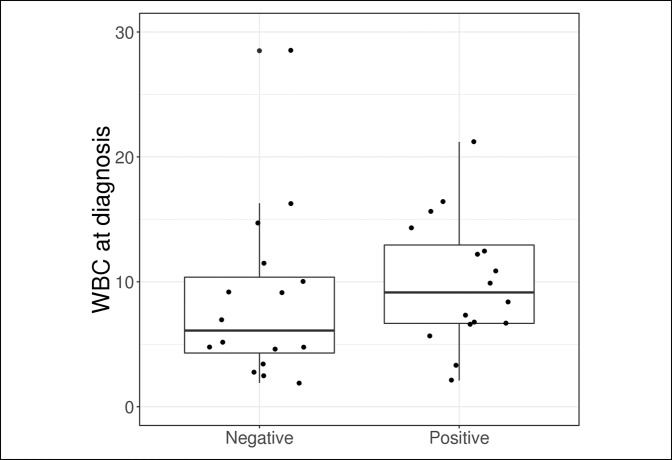
Boxplot of WBC according to the culture result. WBC, white blood cell

**Figure 5:**
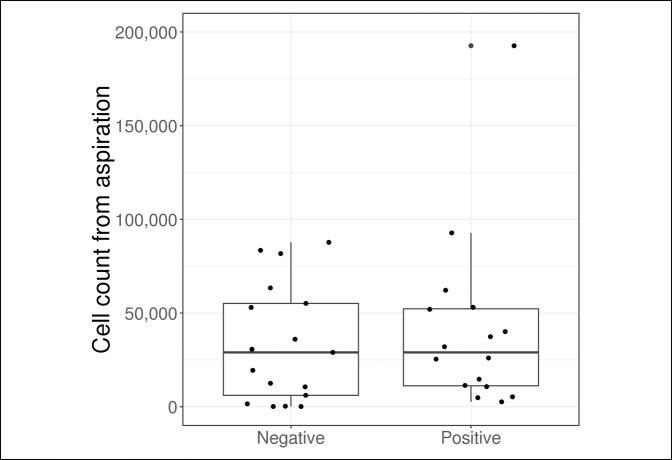
Boxplot of synovial fluid cell count according to the culture result.

Outcome measures are compared in Table 2 and Figures 1–5. The median synovial fluid cell count in the culture-positive cohort was 29,000 cells/mm^3^. Similarly, the culture-negative patients had a median synovial cell count of 29,000 cells/mm^−3^ (*P* = 0.64). Only five patients (31.2%) in the culture-positive cohort had a synovial fluid cell count above the 50,000 cells/mm^−3^ threshold. The synovial fluid cell count was not effective in discriminating between patient groups (culture-positive or culture-negative) with an AUC of 0.55.

**Table 2:** Outcome Measures According to Culture for Septic Arthritis Status (Negative or Positive)

	Negative Culture (N = 17)	Positive Culture (N = 16)	AUC (95% CI)	*P* Value
% Neutrophils	84 (25, 98)	92.5 (23, 99)	0.61 (0.41-0.81)	0.29
ESR at diagnosis	46.0 (2.0, 87.0)	70.0 (20.0, 128.0)	0.71 (0.50-0.92)	0.081
CRP at diagnosis	65.5 (0.5, 245.4)	138.2 (3.5, 399.0)	0.65 (0.42-0.88)	0.22
WBC at diagnosis	6.1 (1.9, 28.5)	9.2 (2.1, 21.2)	0.63 (0.43-0.83)	0.23
Cell count from aspiration	29,000 (122, 87750)	29,000 (2600, 192,667)	0.55 (0.34-0.75)	0.64

AUC = area under the ROC curve, CI = confidence interval, CRP = C-reactive protein, ESR = erythrocyte sedimentation rate, WBC = white blood cell

Continuous variables are summarized with median (range), and categorical variables are summarized with number (%). *P* values less than 0.05 are considered statistically significant and are shown in bold.

Although no statistically significant differences were observed between patients with a positive and negative culture based on laboratory values, ESR at diagnosis was slightly higher in the culture-positive group (median: 70.0 versus 46.0, *P* = 0.081) and was moderately effective in discriminating between patients with a positive and negative culture with an AUC of 0.71. No other laboratory value was found to be helpful in predicting culture-positive patients.

## Discussion

Diagnosing septic arthritis often relies on clinical acumen and a careful review of laboratory data. Although it is important for the clinician to proceed in an expedited fashion, overly aggressive diagnosis may result in patients undergoing unnecessary procedures; accurate diagnosis therefore is paramount.^22^ Although the benchmark for diagnosis remains a positive culture from the affected joint, this is often not practical because culture results may take several days, increasing patient morbidity. It is for this reason that previous literature has established a synovial fluid cell count threshold of 50,000 cells/mm^−3^, as suggestive of a diagnosis of septic arthritis in a native joint in lieu of positive culture results, with lower values suggesting a crystalline or inflammatory arthropathy.^10,14,15,16,17,23^ Not only is the literature to support this cutoff limited, there are little data to support this threshold in the immunosuppressed patient population.^12,20^ The purpose of this study was therefore to retrospectively review our immunosuppressed patients with culture-positive septic arthritis to determine if 50,000 cells/mm^−3^ is a valid threshold in this population.

Previous studies have used varying definitions for septic arthritis.^10,14,15,24,25^ In 1976, Newman^24^ defined septic arthritis based on the following criteria: (1) isolating an organism from the affected joint, (2) isolating an organism from elsewhere in a patient with a clinically swollen, painful joint, (3) histologic or radiologic evidence of infection, or (4) turbid fluid aspirated from the joint. This definition remains popular even today.^10^

In our study, we did not choose to use the septic arthritis definition from Newman, rather only included patients with the gold standard culture-positive septic arthritis. In doing so, we likely did not include all examples of septic arthritis in the immunosuppressed patients at our institution. Our reason for this decision was to limit the chance that we included patients without true septic arthritis and the potential for their data to skew our results. In addition, in our comparison group, we excluded all patients who had a negative culture but had received antibiotics before the aspiration because this may have masked a true infection. Although realizing this is not a perfect control, as evidenced by the fact that some of these patients still underwent a débridement procedure based on clinical evaluation, we think this group's data are useful to contrast against our culture-positive cohort.

There are several previous studies that have attempted to determine the best laboratory tests for accurate diagnosis of septic arthritis.^10,15,26–28^ In a systematic review, Margaretten et al found that synovial fluid cell count and the percentage of polymorphonuclear cells in the aspiration were the most powerful predictors of septic arthritis. They found that as the synovial fluid cell count and percentage of polymorphonuclear cells increased, so too did the likelihood ratio of the patient having septic arthritis.^10^ A meta-analysis by Carpenter et al^26^ similarly found a higher likelihood ratio for septic arthritis, with a higher synovial fluid cell count. Both of these studies reported the highest likelihood ratio for septic arthritis when the synovial fluid cell count was >50,000 cells/mm^−3^.

Contrasting the previous studies, Li et al reported that the 50,000 cells/mm^−3^ threshold only had a sensitivity of 50%. The authors recommended a stricter threshold of 17,500 cells/mm^−3^ to increase the sensitivity of the test and avoid a missed or delayed diagnosis. They recommended using the threshold value to rule out septic arthritis rather than attempt to diagnose it.^15^

Although these previous studies are helpful, none of them address how to handle an immunosuppressed patient. Often these patients are the most challenging to diagnose, and the morbidity from a delayed or missed diagnosis can be magnified in this population. In our study, we found that the median synovial fluid cell count in culture-positive immunosuppressed septic arthritis patients was 29,000 cells/mm^−3^, far lower than the 50,000 cells/mm^−3^ threshold. This value was similar to the patients who were culture-negative and was not found to be effective in predicting culture results. In addition, only five patients (31.2%) with culture-positive septic arthritis had a synovial fluid cell count greater than the 50,000 cells/mm^−3^ threshold. If clinicians only relied on the synovial fluid cell count in this patient population, they would miss the diagnosis in almost 70% of patients based on the currently accepted threshold.

Besides the synovial fluid cell count we also evaluated the serum WBC count, ESR, and CRP. We additionally evaluated the percentage of synovial neutrophils and found that none of these values were helpful in predicting which patients would have positive cultures in the immunosuppressed population. Based on these results we recommend that clinicians have a heightened suspicion for septic arthritis in immunosuppressed patients and rely more on clinical evaluation than the laboratory values to avoid a delayed or missed diagnosis.

There are unavoidable limitations with this study. This was performed as a retrospective review and has all the inherent limitations of this study design. Although this study comprised mostly of septic arthritis involving the knee joint, other joints were included in the analysis, which adds to the complexity of interpreting the results because different joints could have different physiologic responses to infection. Previous studies have however used a similar approach. The main limitation of this study is the small sample size, which is a combined effect of studying a rare patient population and our strict criteria for inclusion into the study. This results in a low power to detect differences between our patient cohorts. A multicenter approach would be needed to overcome this. Despite these limitations, we think these data are valuable because the main focus of the study was to determine if the average cell count in the immunosuppressed patients reached the commonly cited 50,000 cells/mm^−3^ threshold rather than to determine absolute differences between the patient cohorts.

## Conclusion

This study determined that traditional serum and synovial laboratory markers were unable to reliably predict culture-positive septic arthritis in the immunosuppressed patient cohort. Notably, the culture-positive immunosuppressed patients were found to have an average synovial fluid cell count of 29,000 cells/mm^−3^, far less than the often-quoted 50,000 cells/mm^−3^ threshold. We recommend relying more on clinical evaluation than laboratory values for early diagnosis and treatment of septic arthritis in the immunosuppressed patient cohort.
